# Demography when history matters: construction and analysis of second-order matrix population models

**DOI:** 10.1007/s12080-017-0353-0

**Published:** 2017-12-08

**Authors:** Charlotte de Vries, Hal Caswell

**Affiliations:** Institute for Biodiversity and Ecosystem Dynamics, University of Amsterdam, Amsterdam, The Netherlands

**Keywords:** Second-order matrix population model, Individual heterogeneity, Prior stage dependence, Carry over effects, Sensitivity analysis

## Abstract

History matters when individual prior conditions contain important information about the fate of individuals. We present a general framework for demographic models which incorporates the effects of history on population dynamics. The framework incorporates prior condition into the *i*-state variable and includes an algorithm for constructing the population projection matrix from information on current state dynamics as a function of prior condition. Three biologically motivated classes of prior condition are included: prior stages, linear functions of current and prior stages, and equivalence classes of prior stages. Taking advantage of the matrix formulation of the model, we show how to calculate sensitivity and elasticity of any demographic outcome. Prior condition effects are a source of inter-individual variation in vital rates, i.e., individual heterogeneity. As an example, we construct and analyze a second-order model of *Lathyrus vernus*, a long-lived herb. We present population growth rate, the stable population distribution, the reproductive value vector, and the elasticity of *λ* to changes in the second-order transition rates. We quantify the contribution of prior conditions to the total heterogeneity in the stable population of *Lathyrus* using the entropy of the stable distribution.

**Electronic supplementary material:**

The online version of this article (10.1007/s12080-017-0353-0) contains supplementary material, which is available to authorized users.

## Introduction

Every demographic analysis requires a classification of individuals by age, size, developmental stage, physiological condition, or some other variable. These variables describe individual states (*i*-states) such that the fate of an individual depends only on its current state and the environment (Metz 1977; Caswell and John 1992; Metz and Diekmann 1986). This requires the state variable to capture all the aspects of the individual’s history that are relevant to its future fate (Caswell et al. 1972; Caswell 2001). The task of the population modeler is to find an *i*-state variable that successfully captures past history. This is not easy; apparently reasonable and frequently used choices of *i*-states may fail to capture all the relevant information about individual history.

**Table 1:** Terminology used to distinguish the state of an individual, the stage of the life cycle, and the prior condition of the individual

∙	The *state* of an individual is the information necessary to predict the response of an individual to its environment.
∙	The *stage* of an individual refers to a biologically defined category, usually a life cycle stage, which is used to define a (possibly unsuccessful) state variable.
∙	The *condition* of an individual is a flexible term that refers to some function of the prior stage and current stage of the individual; this historical information may be combined with the present stage to obtain a state variable based on current stage and prior condition.

Confronted with this problem, the modeler might choose a completely different *i*-state variable (as plant ecologists did when giving up age-classified demography in favor of size-classified models), or might add a dimension to the state space (as in extending stage-classified models to include both age and stage). Sometimes, however, it might be difficult or impossible to measure the relevant current characteristic, but a proxy for that characteristic might be found in some function of the prior condition of an individual. For example, resource storage can influence vital rates in plants but resource storage can be difficult to measure. However, reproduction in the prior year can be used as a proxy for resource storage in species where reproduction in one year may deplete resource storage and reduce fertility in the following year. If it is not possible to measure resource storage, one might therefore incorporate prior reproductive status into the state variable to improve the model.

A variety of prior conditions which affect vital rates have been found empirically. Reynolds and Burke (2011) found that chestnuts with fast early growth died younger than chestnuts with slow early growth. Warren et al. (2014) found that previous breeding success and current body condition may be among the most important determinants of breeding propensity in female lesser scaup. Rouan et al. (2009) found that choice of next breeding site is affected by both current and prior breeding site in *Branta canadensis*. These examples show that prior condition can be a source of individual variation in vital parameters, i.e., a source of heterogeneity. Some attempts have been made to include this source of heterogeneity into population models, see Pfister and Wang (2005), Ehrlén (2000), and Rouan et al. (2009), but a framework for incorporating general prior conditions into demographic models does not exist and will be presented in this paper.

When constructing a structured population model, the *i*-state variables are used to classify individuals into states in a population vector **n**(t) whose entries give the densities of each state. The population vector is projected forward by a population projection matrix **A**

1 $$\begin{array}{@{}rcl@{}} \mathbf{n}(t + 1) &=& \mathbf{A} \mathbf{n}(t). \end{array} $$

The matrix **A** can be decomposed into a matrix **U**, containing transition probabilities for existing individuals, and a matrix **F**, describing the generation of new individuals by reproduction:

2 $$ \mathbf{A}=\mathbf{U}+\mathbf{F}. $$

If prior conditions influence present dynamics, the vector **n** and the matrices **U**, **F**, and **A** must be modified to account for these influences. Our goal in this paper is to present a systematic method for constructing such models in which individuals are classified by current stage and (very generally defined) prior condition. Because we are considering effects of individual condition at just one prior time, we refer to these as second-order matrix population models. We will present the demographic analysis of such models at the level of the individual, the cohort, and the population, and show how to carry out sensitivity analyses of model results to changes in parameters. As an example, we develop a model and calculate the elasticity of the population growth rate of the herbaceous perennial plant *Lathyrus vernus*.

### Terminology

Our discussion requires careful definitions of terms in order to clarify the way that historical effects are incorporated. We will say that the life cycle is described in terms of *stages* (e.g., size classes). The prior *condition* of an individual is some function of its stage at the prior time and its stage at the current time, and thus incorporates historical information. The combination of current stage and prior condition serves as the individual *state* variable for the analysis. We give an overview of the terminology used in this paper in Table 1.

The prior condition can be any arbitrary function of prior and current stage. Prior condition might be the stage at the prior time, it might be defined by membership in a set of stages at the prior time, or it might be defined as the difference between the current and the prior stages. Suppose for example that stages are defined by size, in the hope that a size classification would be a satisfactory *i*-state variable. It might turn out that historical effects require including size at the prior time in the *i*-state variable. Alternatively, the *i*-state might require information only on membership in a class of sizes (e.g., larger than average or smaller than average); we will refer to these classes of prior stages as equivalence classes. Or, the *i*-state might require information on the change in size between the previous time and the current time, and individuals might be classified by whether they have grown, shrunk, or remained in the same size class.

Models based on prior stage are described in section “Full prior stage dependence,” models based on general functions of prior and current stages are described in section “Prior condition models,” and models based on equivalence classes of prior stage are described in section “Equivalence classes of prior stages.” The matrices, vectors, and mathematical operations used in this paper are listed in Table 2.

**Table 2:** Mathematical notation used in this paper

Quantity	Description	Dimension
$\tilde {\mathbf {U}}$	Prior stage dependent cohort projection matrix	*s*(*s* + 1) × *s*(*s* + 1)
**U**_*i*_	Matrix with transition rates (from current to future state) for individuals with prior stage *i*	*s* × *s*
$\tilde {\mathbf {V}}$	Prior condition dependent cohort projection matrix	*s* *r* × *s* *r*
$\tilde {\mathbf {F}}$	Prior stage dependent fertility matrix	*s*(*s* + 1) × *s*(*s* + 1)
**F**_*i*_	Matrix with fertility rates (from current to future state) for individuals with prior stage *i*	*s* × *s*
$\tilde {\mathbf {G}}$	Prior condition dependent fertility matrix	*s* *r* × *s* *r*
$\tilde {\mathbf {n}}$	Prior stage model population vector $\tilde {\mathbf {n}}$ = vec(**N**)	*s*(*s* + 1) × 1
$\tilde {\mathbf {m}}$	Prior condition model population vector $\tilde {\mathbf {m}}$ = vec(**M**)	*s* *r* × 1
**C**	Matrix relating the prior stage dependent vector $\tilde {\mathbf {n}}$ to the prior condition dependent population vector $\tilde {\mathbf {m}}$	*s* *r* × *s*(*s* + 1)
***ϕ***(*i*,*j*)	Matrix whose (*i*,*j*)th entry indicates the prior condition for an individual that makes an *i* → *j* transition	*s* × *s*
***ϕ*** ^*k*^	Matrix whose (*i*,*j*)th entry is one if ***ϕ***(*i*,*j*) = *k* and zero otherwise, in Matlab notation *ϕ* ^*k*^(*i*,*j*) = (*ϕ* == *k*)	*s* × *s*
**I**_*s*_	Identity matrix	*s* × *s*
**1**_*s*_	Vector of ones	*s* × 1
**e**_*i*_	The *i* th unit vector, with a 1 in the *i* th entry and zeros elsewhere	Various
**E**_*ij*_	A matrix with a 1 in the (*i*,*j*) position and zeros elsewhere	Various
⊗	Kronecker product	
**X**(:,*i*)	Column *i* of matrix **X**	
vec**X**	The vec operator, which stacks the columns of an *m* × *n* matrix X into a *m* *n* × 1 vector	

Dimensions of vectors and matrices are given where relevant; *s* denotes the number of classes in the full second-order model and *r* denotes the number of classes in the reduced second-order model

## Model construction

We will begin by constructing the model in which the prior condition is defined by full information on the prior stage, which we refer to as full prior stage dependence.

### Full prior stage dependence

Creating a prior stage model requires transition and fertility rates to be measured for each possible prior stage. Since newborns do not have a well-defined prior stage in general, an extra prior stage must be added for newborns. If there are *s* current stages, we will label the prior condition of newborns as stage *s* + 1. Individuals are thus classified by their current stage 1,2,…,*s* and their prior stage 1,2,…,*s* + 1. The transition and fertility rates, conditional on prior stage, are given by the matrices **U**_*k*_ and **F**_*k*_:

3 $$\begin{array}{@{}rcl@{}} \mathbf{U}_{k} & s \times s& \text{ Transitions among stages for individuals } \end{array} $$

4 $$\begin{array}{@{}rcl@{}} & & \text{ with prior stage}\, k = 1, \dots, s + 1, \\ \mathbf{F}_{k} & s \times s & \text{ Reproduction by individuals } \end{array} $$

$$\begin{array}{@{}rcl@{}} & & \text{with prior stage}\, k = 1, \dots, s + 1. \end{array} $$

The entries of **U**_*k*_, denoted by $u^{k}_{ij}$, are defined as

5 $$\begin{array}{@{}rcl@{}} u^{k}_{ij}&=& \text{P} \left[j \to i \mid \text{prior stage}=k\right], \end{array} $$

6 $$\begin{array}{@{}rcl@{}} f^{k}_{ij}&=& \text{E} \left[ \text{offspring of stage } i \mid \text{prior stage}=k \right]. \end{array} $$

It is useful to think of the state of the population as described by a two-dimensional array **N** of size *s* × (*s* + 1) 
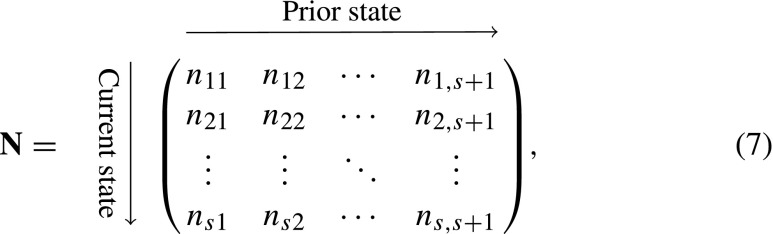
 where *n*_*ij*_ is the number of individuals whose current stage is *i* and whose prior stage was *j*. Ehrlén (2000) suggested that this array could be updated by multiplication with a three-dimensional matrix. However, matrix multiplication can not be used directly to project such a two-dimensional array (this would require tensors). Instead, the two-dimensional array is transformed into a vector by stacking the columns on top of each other:

8 $$ \tilde{\mathbf{n}}(t)=\text{vec}\mathbf{N}(t).  $$

We use the tilde notation to denote vectors and matrices that relate to the prior condition model. The entries in the population vector $\tilde {\mathbf {n}}$ are now ordered first by prior stage and then by current stage: 
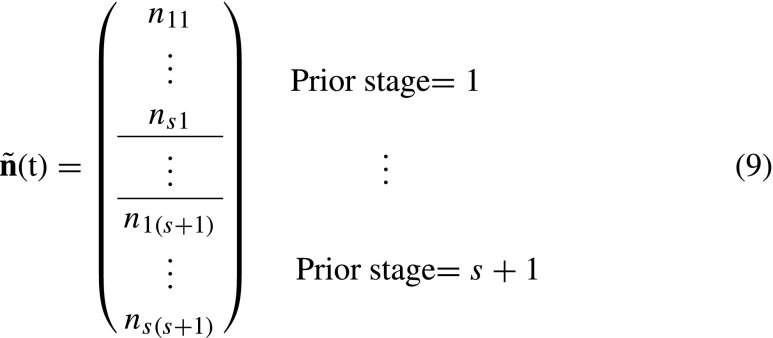


The vector $\tilde {\mathbf {n}}$ is projected by a transition matrix $\tilde {\mathbf {U}}$ and a fertility matrix $\tilde {\mathbf {F}}$. The $\tilde {\mathbf {U}}$ and $\tilde {\mathbf {F}}$ matrices are constructed, respectively, from the set of matrices **U**_*k*_ and **F**_*k*_. The transition matrix $\tilde {\mathbf {U}}$ projects individuals into their next stage while keeping track of their prior stage. The matrix $\tilde {\mathbf {U}}$ is written in terms of block matrices corresponding to the blocks in $\tilde {\mathbf {n}}$ in Eq. 1. In Matlab notation, the transition matrix for a model with *s* = 2 is 
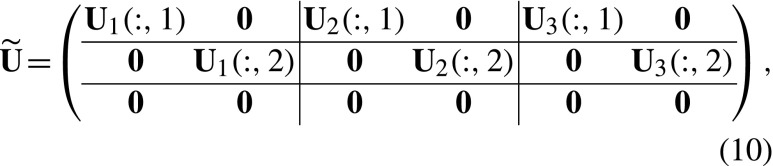
 where **U**_*k*_(:,*i*) refers to the *i* th column of the matrix **U**_*k*_ and **0** is a column vector of size *s* × 1. To understand the structure of $\tilde {\mathbf {U}}$, consider the (1,1) block in the upper left corner. This block projects individuals with prior stage 1 at *t* to prior stage 1 at *t* + 1. The only such individuals have current stage 1 at time *t*. They are projected by column 1 of **U**_1_. All other columns of the (1,1) block of $\tilde {\mathbf {U}}$ are zero. The other blocks of $\tilde {\mathbf {U}}$ are filled similarly.

The blocks in the last row of $\tilde {\mathbf {U}}$ are zero because transitions into the newborn stage are impossible; the matrix $\tilde {\mathbf {F}}$ will fill up this row block. In general, for any number of stages *s*,

11 $$ \mathbf{\widetilde{U}}=\sum\limits_{j = 1}^{s + 1} \sum\limits_{i = 1}^{s} \mathbf{E}_{ij} \otimes \left( \mathbf{e}_{i}^{\top} \otimes \mathbf{U}_{j} \mathbf{e}_{i} \right),  $$

where **e**_*i*_ has size *s* × 1 and **E**_*ij*_ has size (*s* + 1) × (*s* + 1) and has a one in position (*i*,*j*) and zeros elsewhere.

The fertility matrix $\tilde {\mathbf {F}}$ is constructed from the **F**_*i*_. Because individuals are always born into stage *s* + 1, the **F**_*i*_ appear in the last row of blocks in $\tilde {\mathbf {F}}$. For the case with *s* = 2, the fertility matrix is 
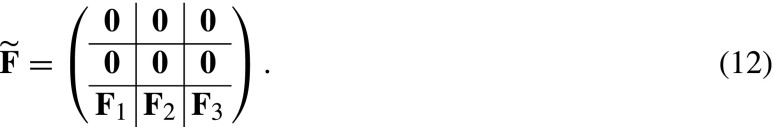
 In general, for any number of stages *s*,

13 $$ \mathbf{\widetilde{F}}=\sum\limits_{k = 1}^{s + 1} \mathbf{E}_{(s + 1)k} \otimes \mathbf{F}_{k},  $$

where **E**_(*s*+ 1)*k*_ has size (*s* + 1) × (*s* + 1) and has a one in position (*s* + 1, *k*) and zeros everywhere else.

Given a set of transition matrices **U**_*i*_ and fertility matrices **F**_*i*_, Eqs. 11 and 13 define the second-order matrices $\tilde {\mathbf {U}}$ and $\tilde {\mathbf {F}}$, and the population projection matrix

14 $$ \tilde{\mathbf{A}}=\tilde{\mathbf{U}}+\tilde{\mathbf{F}}.  $$

These matrices are subject to all the usual demographic analyses, including population growth, population structure, and sensitivity analysis (see section “Case study” for an example).

### Prior condition models

A more general second-order model structure allows transitions and fertility to depend on some function of current and prior stages. We consider the case where this function can be defined as a linear transformation of the full prior stage dependent model, where the population vector is written as $\tilde {\mathbf {m}}$, defined by

15 $$ \tilde{\mathbf{m}}(t)=\textbf{C} \tilde{\mathbf{n}}(t),  $$

for some matrix **C**. An example of such a prior condition is having previously grown, shrunk, or stayed the same size. The matrix **C** maps *i*-states defined by the combination of (current stage × prior stage) to *i*-states defined by the combination of (current stage × prior condition).

Suppose there are *r* distinct prior conditions. The state of the population is now given by a *s* × *r* array **M** 
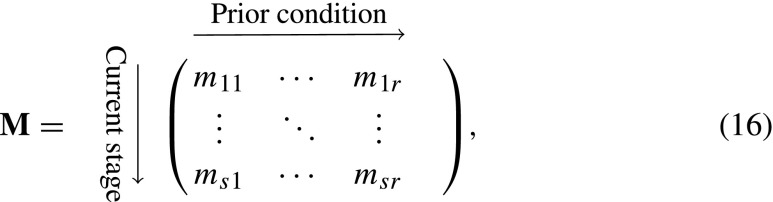
 where *m*_*ij*_ represents the number of individuals whose current stage is *i* and whose prior condition is *j*. As in Eq. 8, the population vector $\tilde {\mathbf {m}}$ is

17 $$ \tilde{\mathbf{m}}(\text{t})=\text{vec}\mathbf{M}(t). $$

The key to the construction of the prior condition model is to derive **C** from the rule defining the prior condition. To do so, define a matrix ***ϕ***

18 $$\begin{array}{@{}rcl@{}} \boldsymbol{\phi}(i,j)&&=\text{prior condition for an individual that makes an}\\\,&&\quad j \to i\, \text{transition.} \end{array} $$

For example, if the *r* = 3 prior conditions are shrinking, stasis, and growth

19 $$ \boldsymbol{\phi}(i,j) = \left\{ \begin{array}{lll} 1 & \text{for } i < j & \text{shrinking}, \\ 2 & \text{for } i=j & \text{stasis}, \\ 3 & \text{for } i>j & \text{growth}. \end{array}\right. $$

Next, we define a set of matrices ***ϕ*** ^*k*^(*i*,*j*), for *k* = 1,…,*r*, given by

20 $$ \boldsymbol{\phi}^{k}(i,j)= \left\{ \begin{array}{lll} 1 & \text{if } \boldsymbol{\phi}(i,j)=k , \\ 0 & \text{otherwise }. \end{array}\right. $$

Given the matrices ***ϕ*** ^*k*^, the matrix **C** is given by

21 $$ \mathbf{C}= {\sum}_{k = 1}^{r} \left( \mathbf{1}^{{\textsf{T}}}_{s + 1}\otimes \mathbf{e}_{k} \otimes \mathbf{I}_{s}\right) \text{diag}\left( \text{vec} \boldsymbol{\phi}^{k}\right),  $$

see Appendix A. To project the new population vector $\tilde {\mathbf {m}}$, we replace the matrices $\tilde {\mathbf {U}}$ and $\tilde {\mathbf {F}}$ with new matrices $\tilde {\mathbf {V}}$ and $\tilde {\mathbf {G}}$, respectively, so that

22 $$ \tilde{\mathbf{m}}(t + 1)=\left( \tilde{\mathbf{V}}+\tilde{\mathbf{G}}\right) \tilde{\mathbf{m}}(t)  $$

The matrix $\tilde {\mathbf {V}}$ describes transitions of extant individuals between the different *i*-states of the prior condition model and the matrix $\tilde {\mathbf {G}}$ describes the production of new individuals in the prior condition model. Substituting (15) into both sides of Eq. 22 yields

23 $$ \mathbf{C}\left( \tilde{\mathbf{U}}+\tilde{\mathbf{F}}\right) \tilde{\mathbf{n}}(t)=\left( \tilde{\mathbf{V}}+\tilde{\mathbf{G}}\right) \mathbf{C}\tilde{\mathbf{n}}(t).  $$

Equation 23 is satisfied if $\tilde {\mathbf {V}}$ and $\tilde {\mathbf {G}}$ satisfy the following equations:

24 $$\begin{array}{@{}rcl@{}} \tilde{\mathbf{V}}\mathbf{C}=\mathbf{C}\tilde{\mathbf{U}} \end{array} $$

25 $$\begin{array}{@{}rcl@{}} \tilde{\mathbf{G}}\mathbf{C}=\mathbf{C}\tilde{\mathbf{F}}. \end{array} $$

In general, the matrix **C** is not square and does not have an inverse. So we use the Moore-Penrose pseudo-inverse **C** ^‡^ (see Abadir and Magnus (2005)) to solve for $\tilde {\mathbf {V}}$ and $\tilde {\mathbf {G}}$:

26 $$\begin{array}{@{}rcl@{}} \tilde{\mathbf{V}}&=&\mathbf{C} \tilde{\mathbf{U}} \mathbf{C}^{\dagger}, \end{array} $$

27 $$\begin{array}{@{}rcl@{}} \tilde{\mathbf{G}}&=&\mathbf{C} \tilde{\mathbf{F}} \mathbf{C}^{\dagger}. \end{array} $$

If **C** is square and non-singular, the pseudo-inverse is the ordinary inverse:

28 $$ \mathbf{C}^{\dagger}=\mathbf{C}^{-1}. $$

If **C** is not square but has linearly independent rows (i.e., has full row rank),

29 $$ \mathbf{C}^{\dagger}=\mathbf{C}^{{\textsf{T}}}(\mathbf{C}\mathbf{C}^{{\textsf{T}}})^{-1}.  $$

If **C** has rows of zeroes (this happens if some combinations of current stage and prior conditions are impossible), then **C** will not have full rank. In this case, **C** ^‡^ is computed from the singular value decomposition, implemented in Matlab with the function pinv(C) and in R with the function Ginv(C). For example, in a size-classified model, it is impossible to be in the smallest size class and to have grown into it from a smaller size class. In such cases, **C** ^‡^ and thus also $\tilde {\mathbf {V}}$ and $\tilde {\mathbf {G}}$ have rows and columns of zeros corresponding to the impossible combinations.

Equations 26 and 27 define the prior condition matrices $\tilde {\mathbf {V}}$ and $\tilde {\mathbf {G}}$ and the population projection matrix

30 $$ \tilde{\mathbf{A}}=\tilde{\mathbf{V}}+ \tilde{\mathbf{G}}. $$

As in Eq. 14, the usual demographic results can be obtained from these matrices.

#### Equivalence classes of prior stages

Equivalence classes of prior stages are a special case of functions of current and prior stage. Equivalence classes are subsets of prior conditions that depend only on the prior stage. For example, individuals in a size-classified model might be categorized into two equivalence classes depending on whether they were previously above or below some threshold size.

The machinery described in the previous section, i.e., Eqs. 21, 26, and 27, can be used to write down the equivalence class model. However, there is an easier way to find the **C** matrix that transforms the full prior stage dependent population vector $\tilde {\mathbf {n}}$ to the equivalence class population vector $\tilde {\mathbf {m}}$. We transform the population matrix **N** in Eq. 1 into the equivalence class population matrix **M** in Eq. 1 by a matrix **B**;

31 $$ \mathbf{M}=\mathbf{N} \mathbf{B}.  $$

The matrix **B** has size (*s* + 1) × *r* and its entries are

32 $$ \mathbf{B}(i,j) = \left\{ \begin{array}{lll} 1 & \text{if stage } i \in \text{equivalence class } j,\\ 0 & \text{otherwise.} \end{array}\right. $$

Applying the vec operator to Eq. 31 gives

33 $$ \tilde{\mathbf{m}}(t)=(\mathbf{B}^{\top}\otimes \mathbf{I}_{s}) \tilde{\mathbf{n}}(t),  $$

where we have used the following result from Roth (1934) that vec**A** **B** **C** = (**C** ^T^ ⊗**A**)vec**B**. Equation 33 is a special case of Eq. 15 with

34 $$ \mathbf{C}=(\mathbf{B}^{\top}\otimes \mathbf{I}_{s}).  $$

Since the rows of **B** ^⊤^ are orthogonal, the matrix **B** ^⊤^ and the matrix (**B** ^⊤^⊗**I**_*s*_) both have full row rank and therefore **C** ^‡^ can be calculated from Eq. 29.

## Sensitivity analysis

Sensitivity analysis provides the effect of changes in any parameter on any model outcome. In general, these computations require derivatives of scalar-, vector-, or matrix-valued functions with respect to scalar-, vector-, or matrix-valued arguments. Matrix calculus is a formalism which enables us to consistently calculate such derivates. For an introduction to matrix calculus, see Abadir and Magnus (2005); for details see Magnus and Neudecker (1988). Ecological applications of sensitivity analysis appear in Caswell (2007, 2009, 2012).

Consider some scalar or vector output of the model, *ξ*, which is computed from $\tilde {\mathbf {U}}$ and $\tilde {\mathbf {F}}$, or from $\tilde {\mathbf {V}}$ and $\tilde {\mathbf {G}}$. The matrices $\tilde {\mathbf {U}}$, $\tilde {\mathbf {F}}$, $\tilde {\mathbf {V}}$, and $\tilde {\mathbf {G}}$ are in turn computed from the matrices **U**_*i*_ and **F**_*i*_. Suppose **U**_*i*_ and **F**_*i*_ depend on a *p* × 1 vector of parameters ***𝜃***, i.e., **U**_*i*_ = **U**_*i*_[***𝜃***], then we use the chain rule to write

35 $$\begin{array}{@{}rcl@{}} \frac{\mathrm{d}\xi}{\mathrm{d} \boldsymbol{\theta}^{\top}}&=& \frac{\mathrm{d}\xi}{\mathrm{d} \text{vec}^{\top} \tilde{\mathbf{U}} } \frac{\mathrm{d}\text{vec} \tilde{\mathbf{U}}}{\mathrm{d} \boldsymbol{\theta}^{\top}}+\frac{\mathrm{d}\xi}{\mathrm{d} \text{vec}^{\top} \tilde{\mathbf{F}} } \frac{\mathrm{d}\text{vec} \tilde{\mathbf{F}}}{\mathrm{d} \boldsymbol{\theta}^{\top}}, \end{array} $$

or

36 $$\begin{array}{@{}rcl@{}} \frac{\mathrm{d}\xi}{\mathrm{d} \boldsymbol{\theta}^{\top}}&=& \frac{\mathrm{d}\xi}{\mathrm{d} \text{vec}^{\top} \tilde{\mathbf{V}} } \frac{\mathrm{d}\text{vec} \tilde{\mathbf{V}}}{\mathrm{d} \boldsymbol{\theta}^{\top}}+\frac{\mathrm{d}\xi}{\mathrm{d} \text{vec}^{\top} \tilde{\mathbf{G}} } \frac{\mathrm{d}\text{vec} \tilde{\mathbf{G}}}{\mathrm{d} \boldsymbol{\theta}^{\top}}. \end{array} $$

The first terms in Eqs. 35 and 36 capture effects through survival and transitions, and the second terms capture effects through fertility. The elasticities, or proportional sensitivities, are given by

37 $$ \frac{\epsilon \xi}{\epsilon \boldsymbol{\theta}^{\top}}= \text{diag}^{-1}(\xi) \frac{\mathrm{d}\xi}{\mathrm{d} \boldsymbol{\theta}^{\top}} \text{diag}(\boldsymbol{\theta}).  $$

The derivatives of $\tilde {\mathbf {U}}$, $\tilde {\mathbf {F}}$, $\tilde {\mathbf {V}}$, and $\tilde {\mathbf {G}}$ with respect to ***𝜃*** depend on how $\tilde {\mathbf {U}}$ and $\tilde {\mathbf {F}}$ depend on the **U**_*i*_ and **F**_*i*_ matrices. Differentiating (11), we obtain

38 $$\begin{array}{@{}rcl@{}} \frac{\mathrm{d} \text{vec} \tilde{\mathbf{U}}}{\mathrm{d} \boldsymbol{\theta}^{\top}}&=& \sum\limits_{j = 1}^{s + 1}\mathbf{Q}^{U}_{j} \ \frac{\mathrm{d}\text{vec} \mathbf{U}_{j}}{\mathrm{d} \boldsymbol{\theta}^{\top}}. \end{array} $$

Each term in the summation captures the effect of ***𝜃*** through one of the **U**_*i*_. The matrix $\mathbf {Q}^{U}_{j}$ is given by

39 $$ \mathbf{Q}^{U}_{j}= \sum\limits_{i = 1}^{s} \!\left( \mathbf{I}_{s + 1} \!\otimes \mathbf{K}_{s,s + 1} \!\otimes \mathbf{I}_{s} \right) \!\left( \text{vec}\left( \mathbf{E}_{ij} \right)\!\otimes \mathbf{I}_{s^{2}} \right)\!\left( \mathbf{E}_{ii} \otimes \mathbf{I}_{s} \right). $$

Similarly, differentiating (13) gives

40 $$\begin{array}{@{}rcl@{}} \frac{\mathrm{d} \text{vec} \tilde{\mathbf{F}}}{\mathrm{d} \boldsymbol{\theta}^{\top}}&=& \sum\limits_{i = 1}^{s + 1}\mathbf{Q}^{F}_{i} \ \frac{\mathrm{d}\text{vec} \mathbf{F}_{i}}{\mathrm{d} \boldsymbol{\theta}^{\top}}, \end{array} $$

where each term captures the effect of ***𝜃*** through one of the **F**_*i*_. The matrix $\mathbf {Q}^{F}_{i}$ is given by

41 $$ \mathbf{Q}^{F}_{i}=\left( \mathbf{I}_{s + 1} \otimes \mathbf{K}_{s,s + 1} \otimes \mathbf{I}_{s} \right)\left( \text{vec} \left( \mathbf{E}_{s + 1,i}\right) \otimes \mathbf{I}_{s^{2}} \right). $$

To calculate the derivatives of the prior condition matrices $\tilde {\mathbf {V}}$ and $\tilde {\mathbf {G}}$, we use the chain rule to write

42 $$\begin{array}{@{}rcl@{}} \frac{\mathrm{d} \text{vec} \tilde{\mathbf{V}}}{\mathrm{d} \boldsymbol{\theta}^{\top}}&=& \frac{\mathrm{d} \text{vec} \tilde{\mathbf{V}}}{\mathrm{d} \text{vec} \tilde{\mathbf{U}}} \frac{\mathrm{d} \text{vec} \tilde{\mathbf{U}}}{\mathrm{d} \boldsymbol{\theta}^{\top}}, \end{array} $$

43 $$\begin{array}{@{}rcl@{}} \frac{\mathrm{d} \text{vec} \tilde{\mathbf{G}}}{\mathrm{d} \boldsymbol{\theta}^{\top}}&=& \frac{\mathrm{d} \text{vec} \tilde{\mathbf{G}}}{\mathrm{d} \text{vec} \tilde{\mathbf{F}}} \frac{\mathrm{d} \text{vec} \tilde{\mathbf{F}}}{\mathrm{d} \boldsymbol{\theta}^{\top}}. \end{array} $$

Differentiating Eqs. 26 and 27, we obtain

44 $$\begin{array}{@{}rcl@{}} \frac{\mathrm{d}\text{vec} \tilde{\mathbf{V}}}{\mathrm{d} \text{vec} \tilde{\mathbf{U}}}&=& \left( \mathbf{C}^{\dagger}\right)^{\top} \otimes \mathbf{C}, \end{array} $$

45 $$\begin{array}{@{}rcl@{}} \frac{\mathrm{d}\text{vec} \tilde{\mathbf{G}}}{\mathrm{d} \text{vec} \tilde{\mathbf{F}}}&=& \left( \mathbf{C}^{\dagger}\right)^{\top} \otimes \mathbf{C}. \end{array} $$

Thus,

46 $$\begin{array}{@{}rcl@{}} \frac{\mathrm{d} \text{vec} \tilde{\mathbf{V}}}{\mathrm{d} \boldsymbol{\theta}^{\top}}&=&\left[ \left( \mathbf{C}^{\dagger}\right)^{\top} \otimes \mathbf{C}\right] \frac{\mathrm{d} \text{vec} \tilde{\mathbf{U}}}{\mathrm{d} \boldsymbol{\theta}^{\top}}, \end{array} $$

47 $$\begin{array}{@{}rcl@{}} \frac{\mathrm{d} \text{vec} \tilde{\mathbf{G}}}{\mathrm{d} \boldsymbol{\theta}^{\top}}&=& \left[ \left( \mathbf{C}^{\dagger}\right)^{\top} \otimes \mathbf{C}\right] \frac{\mathrm{d} \text{vec} \tilde{\mathbf{F}}}{\mathrm{d} \boldsymbol{\theta}^{\top}}. \end{array} $$

Equations 38 and 40 are substituted into Eq. 35. Equations 46 and 47 are substituted into Eq. 36. All that remains is to calculate the dependence of *ξ* on $\tilde {\mathbf {U}}$ and $\tilde {\mathbf {F}}$ (or $\tilde {\mathbf {V}}$ and $\tilde {\mathbf {G}}$), and the dependence of **U**_*i*_ and **F**_*i*_ on the parameter *𝜃*. These items are specific to the question under consideration, so we provide an example in the next section.

## Case study

As an example, we apply the second-order formalism to a fully prior stage dependent model of a perennial plant. We will construct the model and calculate the population growth rate, the stable population vector, the reproductive value vector, and the elasticity of the population growth rate *λ* to proportional changes in the demographic rates.

Our analysis is based on a study by Ehrlén (2000) of *Lathyrus vernus*, a long-lived herb native to forest margins and woodlands in central and northern Europe and Siberia. Ehrlén classified individuals into seven stages: seed (SD), seedling (SL), very small (VS), small (SM), large vegetative (VL), flowering (FL), and dormant (DO). Ehrlén constructed two matrix models: a first-order model with no historical effects and a second-order model. We construct our second-order model from the transition and fertility rates calculated by Ehrlén (2000) (their Table 2).

We introduce a special prior stage for newborns; the second-order model therefore has a total of 7 × 8 = 56 states. We used Ehrlén (2000)’s demographic rates to construct a transition matrix **U**_*i*_ and a fertility matrix **F**_*i*_ for each of the eight prior stages. In Table 3, the transition matrix for individuals who were previously in stage SM is shown as an example. The first two columns of this matrix are zero because small plants can not go back to being seeds or seedlings. All eight transition and fertility matrices are in the Supplementary Material.

**Table 3:**
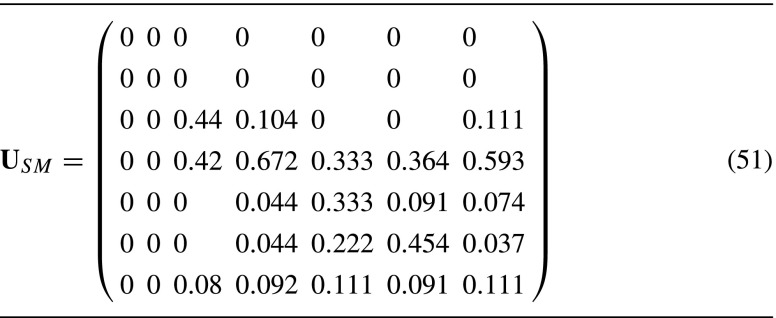
The transition matrix for *Lathyrus vernus* individuals with prior stage small (SM)

The columns in this matrix represent transitions out of the following current stages from left to right: seed (SD), seedling (SL), very small (VS), small (SM), vegetative large (VL), flowering (FL), and dormant (DO)

We constructed the projection matrix, $\tilde {\mathbf {A}}$, from the **U**_*i*_ and **F**_*i*_ matrices using Eqs. 11 and 13. The resulting 56 × 56 matrices are available in the Supplementary Material. The population growth rate *λ* is given by the dominant eigenvalue of $\tilde {\mathbf {A}}$,

48 $$ \lambda= 0.985. $$

**Fig. 1:**
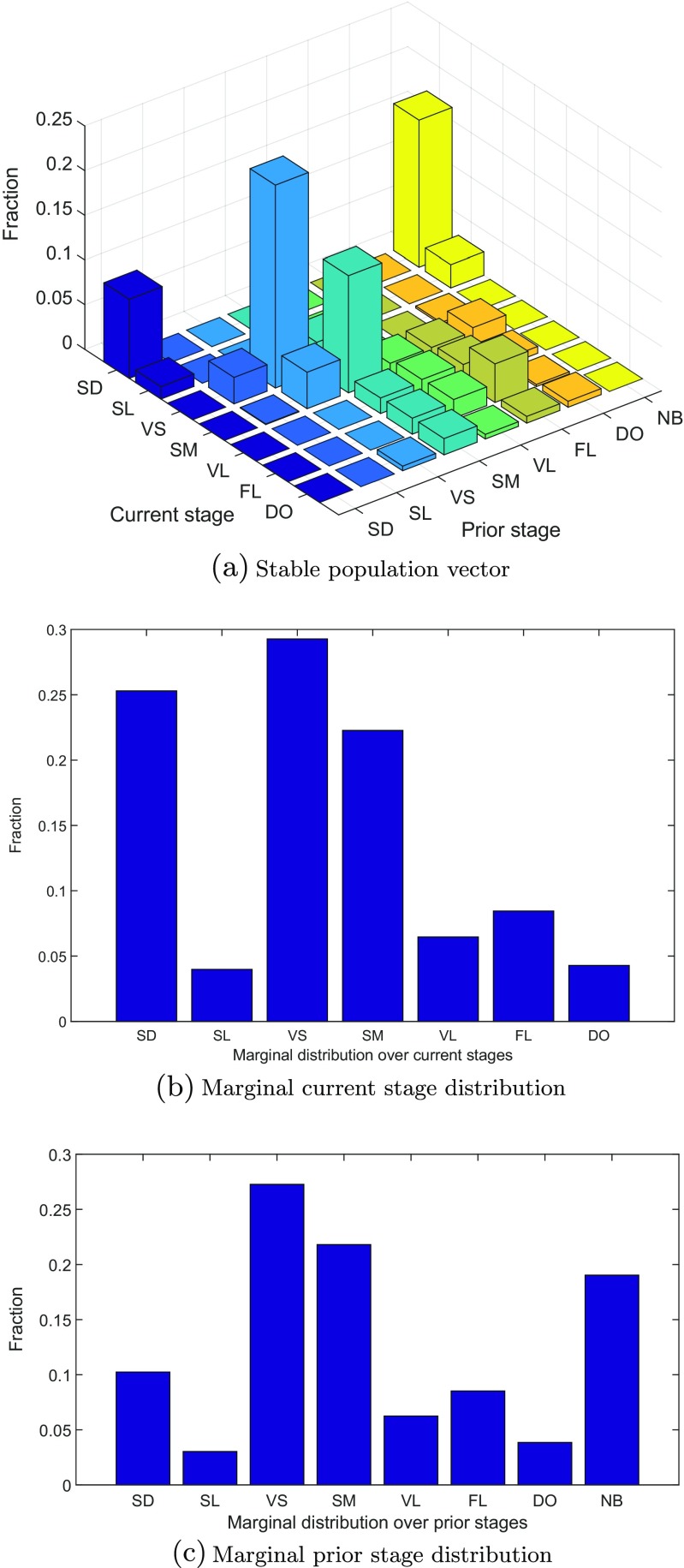
**a** Stable current stage × prior stage distribution. **b** Marginal current stage distribution. **c** Marginal prior stage distribution. Notation: seed (SD), seedling (SL), very small (VS), small (SM), vegetative large (VL), flowering (FL), dormant (DO), and newborn prior (NB)

**Fig. 2:**
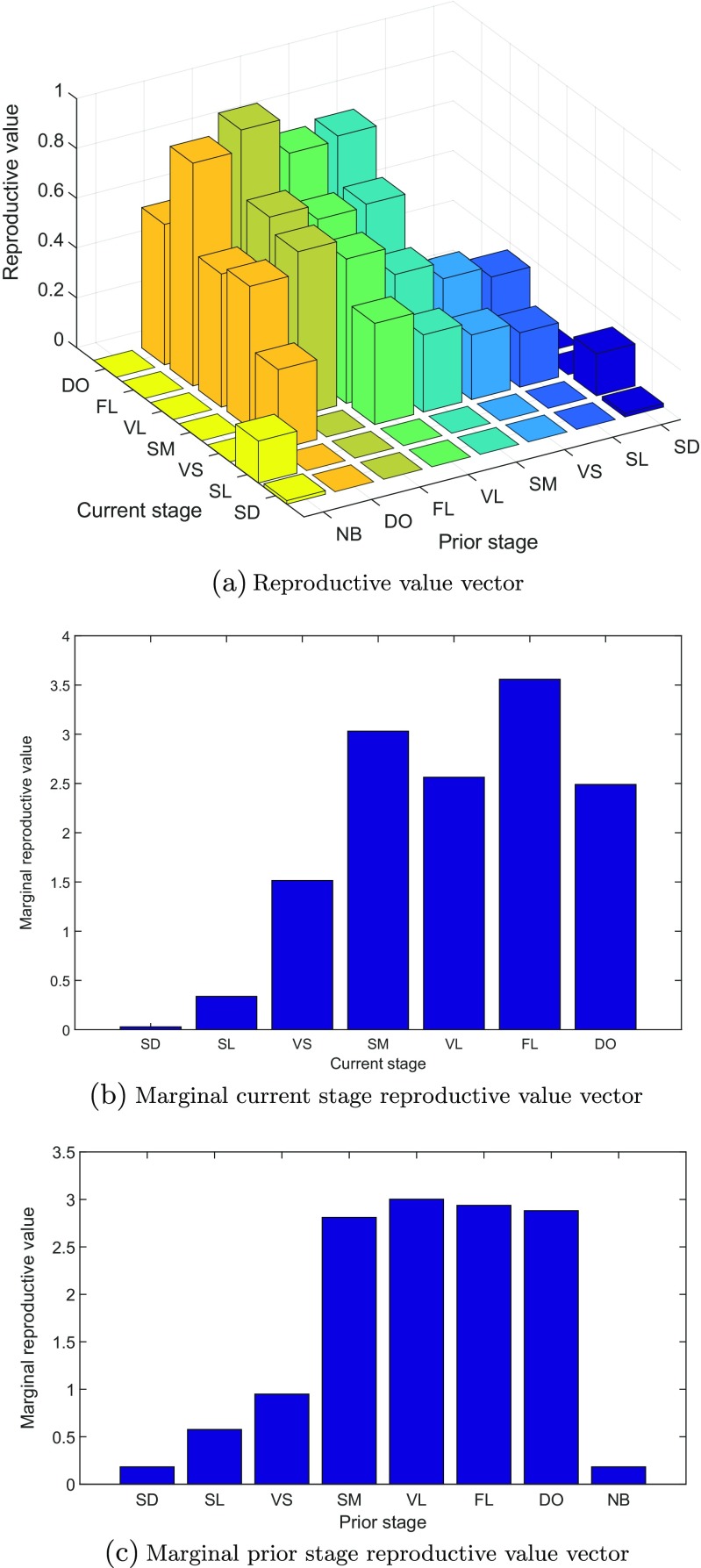
**a** Reproductive value vector for each current stage × prior stage combination. **b** Marginal current stage reproductive value vector. **c** Marginal prior stage reproductive value vector. Notation: seed (SD), seedling (SL), very small (VS), small (SM), vegetative large (VL), flowering (FL), dormant (DO), and newborn prior (NB)

This agrees with the value reported for the second-order model by Ehrlén (2000). Ehrlén also fitted a first-order model to the same data and found a value just above one (*λ* = 1.010). The stable population vector, $\tilde {\mathbf {w}}$, and the reproductive value vector, $\tilde {\mathbf {v}}$, which are displayed in Figs. 1 and 2, are the right and left eigenvectors of $\tilde {\mathbf {A}}$, respectively. The entries of the stable population vector are denoted by $\tilde {w}_{ij}$ for individuals with current stage *i* and prior stage *j*, analogously to the entries of the population vector $\tilde {\mathbf {n}}$ in Eq. 1. The entries of the marginal stable *current* stage distribution, **w** ^*c*^, are given by

49 $$ {w^{c}_{i}}= {\sum}_{j = 1}^{r} \tilde{w}_{ij}  $$

and are shown in Fig. 1b. Similarly, the entries of the marginal stable *prior* stage distribution, **w** ^*p*^, are given by

50 $$ {w^{p}_{j}}= {\sum}_{i = 1}^{s} \tilde{w}_{ij}  $$

and are shown in Fig. 1c.

Individuals with the same current stage have different vital rates if they differ in prior stage and this heterogeneity due to prior stage affects the population growth rate *λ*. To quantify the relative effect of the different prior stage dependent transition matrices on the population growth rate, we calculate the elasticity of the population growth rate, *λ*, to changes in the **U**_*i*_ matrices

52 $$ \frac{\epsilon \lambda}{\epsilon \text{vec}^{\top} \mathbf{U}_{i} }, \qquad i = 1,\ldots,8. $$

Substituting *λ* for *ξ* in Eq. 37 yields

53 $$ \frac{\epsilon \lambda}{\epsilon \text{vec}^{\top} \mathbf{U}_{i} }=\frac{1}{\lambda} \frac{\mathrm{d} \lambda}{\mathrm{d} \text{vec}^{\top} \mathbf{U}_{i}} \text{diag} \left( \text{vec} \mathbf{U}_{i}\right).  $$

As shown in Caswell (2001),

54 $$ \frac{\mathrm{d} \lambda}{\mathrm{d}\text{vec}^{\top} \mathbf{U}_{i}}= \mathbf{w}_{i}^{{\textsf{T}}} \otimes \mathbf{v}_{i}^{{\textsf{T}}}, $$

where **w**_*i*_ and **v**_*i*_ are the right and left eigenvectors of **U**_*i*_, respectively, scaled so that

55 $$\begin{array}{@{}rcl@{}} \mathbf{v}_{i}^{{\textsf{T}}} \mathbf{w}_{i}&= 1 \text{ for all } i, \end{array} $$

56 $$\begin{array}{@{}rcl@{}} \mathbf{1}^{{\textsf{T}}} \mathbf{w}_{i}&= 1 \text{ for all } i. \end{array} $$

**Fig. 3:**
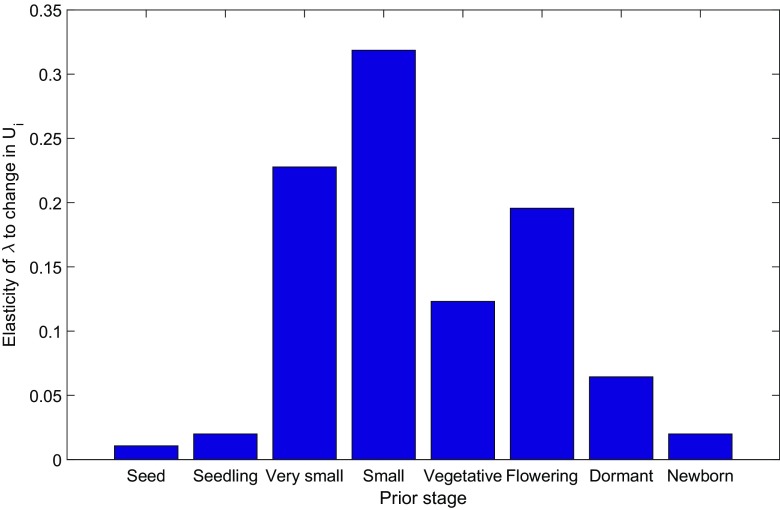
The elasticity of *λ* to a proportional change in all of the **U**_*i*_

We sum the entries of Eq. 53 to get the elasticity of *λ* to a proportional change in all of the entries of **U**_*i*_. The results are shown in Fig. 3. We note that there are large differences among prior stages. Proportional changes in the demography of individuals with prior stages seed, seedling, or newborn have little effect on *λ*. Proportional changes in individuals who were small vegetative at the prior time have effects an order of magnitude larger.

## Discussion

When does history matter? Population models are based on *i*-state variables chosen by some mixture of biological intuition, tradition, practical limitations, and formal statistical analyses. Even after a careful choice of *i*-state, it may happen that individual prior conditions contain important information about the fate of individuals. In such cases, history matters, and the methods presented here solve the problem of how to incorporate information about it into matrix models.

Why stop at second-order effects, what about the effect of the prior condition at *t* − 2, *t* − 3, etc.? It is theoretically possible to extend the framework presented here to include dependence on higher-order effects. However, if the *i*-state variable is such that third- or even fourth-order historical effects are important, it might better to reconsider the choice of *i*-state variable instead of including ever increasing historical dependence.

Statistical tests for second-order effects in longitudinal data using log-linear models have been developed by Bishop et al. (1975) and Usher (1979). Time series of longitudinal data are needed to perform these tests as well as for the subsequent estimation of a prior condition dependent model. Capture–mark–recapture analyses for prior stage dependent models have been developed by Pradel (2005) and Cole et al. (2014).

Incorporating individual history requires a decision about what aspects of the prior condition are important. We have presented three biologically motivated cases: prior condition as the prior stage, prior condition as an arbitrary linear function of current and prior stages, and prior condition as an equivalence class of prior stages. In each case, the necessary information is a set of fertility and survival/transition matrices for each prior stage. The resulting models use block-structured matrices to project a vector of stages within prior conditions. These matrices can be used to calculate all the usual demographic outcomes. Because the matrices are carefully constructed from **U**_*i*_ and **F**_*i*_, they can be subjected to sensitivity analysis. It is straightforward to calculate the sensitivity and elasticity of any model outcome to any parameters affecting the vital rates.

Prior condition effects are more than just a convenient tool in constructing *i*-states for population models. They are a biologically real source of inter-individual variation. The importance of individual variation in vital parameters to ecological processes has become increasingly evident in recent years (Bolnick et al. 2011; Valpine et al. 2014; Caswell 2014; Vindenes and Langangen 2015; Steiner and Tuljapurkar 2012; Cam et al. 2016). Ignoring individual variation in vital parameters, also referred to as individual heterogeneity, can have important consequences for the demographic outcomes and subsequent conclusions; see for example Vaupel et al. (1979), Rees et al. (2000), Fujiwara and Caswell (2001), Vindenes and Langangen (2015), and Cam et al. (2016). In his study of *Lathyrus*, Ehrlén (2000) found that including heterogeneity due to prior stage had only a small effect on *λ*, although it was sufficient to cause the population to decrease rather than increase.

How much heterogeneity is introduced by the prior condition effects in the *Lathyrus* example? Observations of individual plants can identify their current stage, but not their prior condition. The stable structure, $\tilde {\mathbf {w}}$ (Fig. 1), shows the joint probability distribution over current and prior stages, and the amount of heterogeneity in the stable population can be calculated from the entropy of this distribution. The entropy of the joint current × prior stage distribution is

57 $$ \text{H}(p,c)= - {\sum}_{i,j} w_{ij} \ln \left( w_{ij} \right)= 2.61. $$

This measures the overall heterogeneity in the stable population structure. The heterogeneity in the marginal current stage distribution, **w** ^*c*^ [Eq. 49], is

58 $$ \text{H}(c)= - {\sum}_{i = 1}^{s} {w^{c}_{i}} \ln \left( {w^{c}_{i}} \right)= 1.69. $$

This is the observable heterogeneity in current stage. The heterogeneity contributed by the unobservable prior stage, taking into account the relationship between current and prior stage, is

59 $$ \text{H}\left( p | c\right)= \text{H}(p,c)-\text{H}(c)= 0.92,  $$

Khinchin (1957). Thus, in this example, the prior stage contributes about 35% of the total heterogeneity in the stable population.

In this paper, we have considered only linear models in constant environments. However, models could easily be constructed to include density effects, by making the **U**_*i*_ and **F**_*i*_ functions of density. Periodic or stochastic models could be constructed by making the **U**_*i*_ and/or the **F**_*i*_ appropriate functions of time. The analysis of the resulting models may pose interesting challenges and is an open research problem for population ecology.

### Electronic supplementary material

Below is the link to the electronic supplementary material.

(PDF 87.8 KB)

(MAT 2.08 bytes)

## Appendix A: Derivation of the matrix **C**

In this appendix, we will derive the matrix **C** in Eq. 21, which transforms the fully prior stage dependent population vector, $\tilde {\mathbf {n}}$, into the prior condition dependent population vector, $\tilde {\mathbf {m}}$,

60 $$ \tilde{\mathbf{m}}(t)=\mathbf{C} \tilde{\mathbf{n}}(t) , $$

as in Eq. 15. Recall that we defined the matrix ***ϕ*** with elements

61 $$\begin{array}{@{}rcl@{}} \boldsymbol{\phi}(i,j)&&=\text{prior condition for an individual that makes an}\\&&\quad j \to i \text{transition,} \end{array} $$

and we defined a set of indicator matrices ***ϕ*** ^*k*^, for *k* = 1,…,*r*, given by

62 $$ \boldsymbol{\phi}^{k}(i,j)= \left\{ \begin{array}{lll} 1 & \text{if } \boldsymbol{\phi}(i,j)=k , \\ 0 & \text{otherwise }. \end{array}\right. $$

In Appendix B, we show a few examples of ***ϕ*** matrices. Recall furthermore the matrix

where *n*_*i**j*_ is the number of individuals whose current stage is *i* and whose *prior stage* was *j*, and the matrix

where *m*_*i**j*_ is the number of individuals whose current stage is *i* and whose *prior condition* was *j*. Finally, recall that the population vectors are given by

65 $$\begin{array}{@{}rcl@{}} \tilde{\mathbf{n}}(t)&=&\text{vec}\mathbf{N}(t), \end{array} $$

66 $$\begin{array}{@{}rcl@{}} \tilde{\mathbf{m}}(t)&=&\text{vec}\mathbf{M}(t). \end{array} $$

Using the above defined matrices ***ϕ*** ^*k*^ and **N**, the matrix **M** can be written as

67 $$ \mathbf{M}={\sum}_{k = 1}^{r} \left( \boldsymbol{\phi}^{k} \circ \mathbf{N} \right) \left( \mathbf{1}_{s + 1}\otimes \mathbf{e}_{k}^{{\textsf{T}}}\right). $$

The first term in the product is a matrix containing the densities of individuals in all entries of **N** that correspond to prior condition *k*, and zeros elsewhere. The second term in the product adds the densities across each row of the matrix ***ϕ*** ^*k*^ ∘**N** and puts these elements in the *k* th column of **M**. Taking the vec of both sides of this equation gives

68 $$\begin{array}{@{}rcl@{}} \tilde{\mathbf{m}} &=& {\sum}_{k = 1}^{r} \left( \mathbf{1}_{s + 1}^{{\textsf{T}}}\otimes \mathbf{e}_{k} \otimes \mathbf{I}_{s}\right) \text{vec}\left( \boldsymbol{\phi}^{k} \circ \mathbf{N}\right), \end{array} $$

69 $$\begin{array}{@{}rcl@{}} &=& {\sum}_{k = 1}^{r} \left( \mathbf{1}^{{\textsf{T}}}_{s + 1}\otimes \mathbf{e}_{k} \otimes \mathbf{I}_{s}\right) \text{diag}\left( \text{vec} \boldsymbol{\phi}^{k}\right) \tilde{\mathbf{n}}, \end{array} $$

where we have used vec**A** **B** **C** = (**C** ^T^ ⊗**A**)vec**B** (Roth 1934) and vec(**A** ∘**B**) = diag(vec**A**)vec(**B**). Comparison with Eq. 60 shows that

70 $$ \mathbf{C}= {\sum}_{k = 1}^{r} \left( \mathbf{1}^{{\textsf{T}}}_{s + 1}\otimes \mathbf{e}_{k} \otimes \mathbf{I}_{s}\right) \text{diag}\left( \text{vec} \boldsymbol{\phi}^{k}\right).  $$

## Appendix B: Examples of ***ϕ*** and **C** matrices

### B.1 Examples of *ϕ* matrices

In this appendix, we show a few examples of the matrix ***ϕ***.

(A) When *prior condition = prior stage*, then
  71 $$ \boldsymbol{\phi}(i,j)=j \text{ for all i.} $$
  For example, when *s* = 3, ***ϕ*** is
(B) When *prior condition = equivalence classes of prior stages*, then
  74 $$ \boldsymbol{\phi}(i,j)=\text{function}(j) $$
  For example, for *s* = 3 and for the case of the equivalence classes *j* = 1 and *j* > 1, we get
  75 $$ \boldsymbol{\phi}(i,j) =\left\{ \begin{array}{ll} 1 & \text{for } j = 1 \\ 2 & \text{for } j = 2,3 \end{array}\right. $$
(C) When *prior condition = shrinkage, stasis, or growth*, then
  76 $$ \boldsymbol{\phi}(i,j) = \left\{ \begin{array}{lll} 1 & \text{for } i < j & \text{shrinkage} \\ 2 & \text{for } i=j & \text{stasis} \\ 3 & \text{for } i>j & \text{growth} \end{array}\right.  $$
  For example, for *s* = 3, ***ϕ*** is
(D) General more complicated growth conditions. For example, suppose the prior condition reflects the amount of growth, so that
  78 $$ \boldsymbol{\phi}(i,j) = i-j +s. $$
  For *s* = 4, this results in the following ***ϕ***

### B.2 An example **C** matrix

We consider the example in Eqs. 76 and 77 with three size classes and three growth classes corresponding to growth, shrinkage, and stasis. For simplicity, we consider a cohort model with no fertility and therefore no special prior condition for newborns. The matrices ***ϕ*** ^*k*^ are

80 $$\begin{array}{@{}rcl@{}} \boldsymbol{\phi}^{1} &=& \left( \begin{array}{ccc} 0 & 1 & 1 \\ 0 & 0 & 1 \\ 0 & 0 & 0 \end{array} \right),\end{array} $$

81 $$\begin{array}{@{}rcl@{}}\boldsymbol{\phi}^{2} &=& \left( \begin{array}{ccc} 1 & 0 & 0 \\ 0 & 1 & 0 \\ 0 & 0 & 1 \end{array} \right), \end{array} $$

82 $$\begin{array}{@{}rcl@{}}\boldsymbol{\phi}^{3}&=& \left( \begin{array}{ccc} 0 & 0 & 0 \\ 1 & 0 & 0 \\ 1 & 1 & 0 \end{array} \right). \end{array} $$

Substituting these into Eq. 70, and letting the sum range from 1 to 3 only, gives the matrix **C** as

The transition matrix for the prior condition model, $\tilde {\mathbf {V}}$, is given by Eq. 26 and requires the pseudo-inverse of the matrix **C**, which can be found using pinv(C) in Matlab or Ginv(C) in R. Substitution of the pseudo-inverse of **C** into Eq. 26 and a few lines of algebra result in  This matrix captures the complicated dependence of transitions on both current stage and prior condition. The placement of the weighted sums of transition probabilities from the **U**_*i*_ matrices would be challenging without the methodology presented here.
