# Supplementary material for: Demography when history matters: construction and analysis of second-order matrix population models
Source: Theor Ecol. 2017 Dec 8;11(2):129–40. doi: 10.1007/s12080-017-0353-0 (PMC6445492; doi:10.1007/s12080-017-0353-0)
Supplement: Supplementary file 1 — (PDF 87.8 KB) [file 12080_2017_353_MOESM1_ESM.pdf]

Read me file for matlab data file `Ui_and_Fi.mat`

October 16, 2017

The `Matlab` data file `Ui_and_Fi.mat` contains the arrays **U** and **F**. These contain the matrices used in the example calculation for *Lathyrus vernus*. Each is a three-dimensional array of dimensions 7×7×8. The arrays are constructed such that

$$\mathbf{U}(:,:,i) = \mathbf{U}_i, \tag{1}$$

i.e. $\mathbf{U}(:,:,i)$ contains the survival and transition rates for individuals with prior stage i. Equivalently,

$$\mathbf{F}(:,:,i) = \mathbf{F}_i. \tag{2}$$
